# Comparative Effects of Acupressure at Local and Distal Acupuncture Points on Pain Conditions and Autonomic Function in Females with Chronic Neck Pain

**DOI:** 10.1155/2011/543291

**Published:** 2010-09-23

**Authors:** Takako Matsubara, Young-Chang P. Arai, Yukiko Shiro, Kazuhiro Shimo, Makoto Nishihara, Jun Sato, Takahiro Ushida

**Affiliations:** ^1^Department of Rehabilitation, Faculty of Health Sciences, Nihon Fukushi University, 26-2 Higashihaemicho, Handa, Aichi 475-0012, Japan; ^2^Multidisciplinary Pain Centre, School of Medicine, Aichi Medical University, Aichi 480-1195, Japan; ^3^Department of Rehabilitation, Faculty of Health Sciences, Nagoya Gakuin University, Aichi 480-1298, Japan; ^4^Futuristic Environmental Simulation Center, Research Institute of Environmental Medicine, Nagoya University, Nagoya 464-8601, Japan

## Abstract

Acupressure on local and distal acupuncture points might result in sedation and relaxation, thereby reducing chronic neck pain. The aim was to investigate the effect of acupressure at local (LP) and distal acupuncture points (DP) in females with chronic neck pain. Thirty-three females were assigned to three groups: the control group did not receive any stimuli, the LP group received acupressure at local acupuncture points, GB 21, SI 14 and SI 15, and the DP group received acupressure at distal acupuncture points, LI 4, LI 10 and LI 11. Verbal rating scale (VRS), Neck Disability Index (NDI), State-Trait Anxiety Inventory (STAI), muscle hardness (MH), salivary alpha-amylase (sAA) activity, heart rate (HR), heart rate variability (HRV) values and satisfaction due to acupressure were assessed. VRS, NDI, STAI and MH values decreased after acupressure in the LP and the DP group. HR decreased and the power of high frequency (HF) component of HRV increased after acupressure in only the LP group. Although acupressure on not only the LP but also the DP significantly improved pain conditions, acupressure on only the LP affected the autonomic nervous system while acupuncture points per se have different physical effects according to location.

## 1. Introduction

Chronic neck pain is a very common symptom especially in females. In general, neck pain is felt as a dull pain, stiffness, or discomfort along the trapezius muscles and the muscles around the scapulae [1]. Common treatment for chronic neck pain consists of medication, trigger point injection, massage, and other physical therapies and patient education [2]. Massage therapy applied on the tender points is popular in patients with chronic neck pain and provides the patients not only with comfort during and immediately after it but also with various side effects such as discomfort/soreness, tiredness/fatigue, and headache afterwards [3]. Recently, alternative therapies such as acupuncture and acupressure have been increasingly sought. Acupressure is a noninvasive and safe technique, which is manipulated with the fingers instead of needles on the traditional acupuncture points, and has been shown to be effective in pain relief, sedation, and relaxation [4, 5]. Tender points located on the trapezius muscles are consistent with local acupuncture points such as “*Jianjing*” (GB 21), “*Jianwaishu*” (SI 14), and “*Jianzhongshu*” (SI 15) and are applied to massage therapy in patients with chronic neck pain. On the other hand, distal traditional acupuncture points, “*Hegu*” (LI 4), “*Shousanli*” (LI 10), and “*Quchi*” (LI 11), are contained in the Large Intestine Meridian of Hand-Yangming and are suggested to be the points for improving neck-shoulder-arm disorders in the Chinese/Japanese traditional medicine.

Chronic pain influences the autonomic nervous system. For example, sympathetic hyperactivation was shown in fibromyalgia (FM) [6], low back pain [7], whiplash associated disorders [8], and migraine [9]. Furthermore, a study showed functional change of the sympathetic nervous system in workers with chronic neck pain [10]. This abnormality in the sympathetic nervous system might generate and sustain chronic pain [11]. Several reports showed that acupuncture and acupressure on the traditional acupuncture points influence the autonomic nervous system [4, 5, 11, 12]. That is, these procedures could modulate the activities of the sympathetic and parasympathetic nerves.

Autonomic nervous function is known to be reflected in heart rate variability. Rhythmic components of HRV can be quantitatively assessed by means of power spectral analysis. HRV is a reliable and noninvasive tool, used to assess autonomic nervous system regulation of the heart [4, 5]. Frequency fluctuations in low frequency of 0.04–0.15 Hz (LF) component of HRV are considered markers of sympathetic and parasympathetic nerve activities, and high frequencies of 0.15–0.4 Hz (HF) component fluctuation of HRV are considered as a marker of parasympathetic nerve activity. Thus, the LF/HF ratio is considered to be an index of sympathetic nerve activity. 

We hypothesized that acupressure not only on the tender points/local acupuncture points, “*Jianjing*” (GB 21), “*Jianwaishu*” (SI 14), and “*Jianzhongshu*” (SI 15), but also on the distal acupuncture points, “*Hegu*” (LI 4), “*Shousanli*” (LI 10), and “*Quchi*” (LI 11), could induce sedation, thereby reducing pain, muscle tone, and disability and changing autonomic nervous activity in subjects with chronic neck pain. In the present study, we therefore investigated what effect pressure applied on the local and distal acupuncture points had on the pain conditions and HRV in females with chronic neck pain.

## 2. Methods

### 2.1. Subjects

After obtaining approval from the ethics committee of Nihon Fukushi University and written informed consent, 33 female subjects who complained of chronic neck pain participated in the present study. The subjects were randomly allocated to three groups. The exclusion criteria were menstruation, cardiovascular or neurological disease, or administration of sedatives, analgesic, or other medication.

### 2.2. Group, Administration, and Measurements

**Figure 1:**
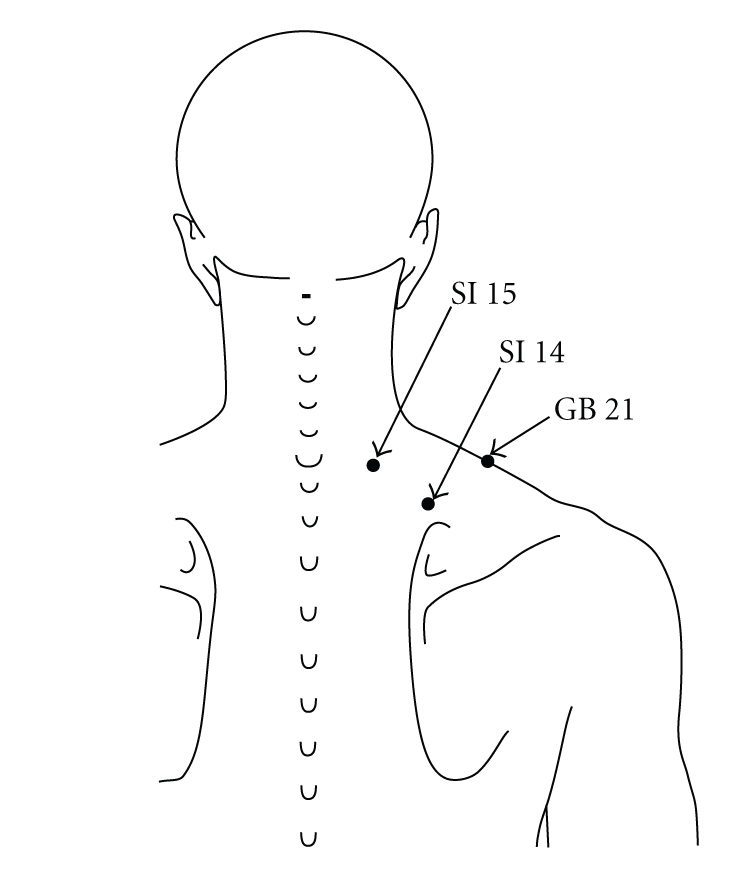
Local acupuncture points/tender points. “*Jianjing*” (GB 21) is located at the highest point on the shoulder and at the midpoint of the line which connects the prominent vertebra and the acromion. “*Jianwaishu*” (SI 14) is located directly above the superior angle of scapula, at 5-6 cm lateral from the posterior midline and below the spinous process of the first thoracic vertebra. “*Jianzhongshu*” (SI 15) is located on the back, at 3-4 cm lateral from the posterior midline and below the spinous process of the seventh cervical vertebra.

**Figure 2:**
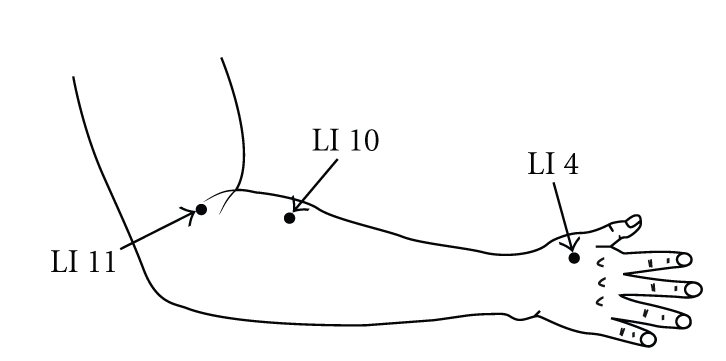
Distal acupuncture point. “*Hegu*” (LI 4) is the most important analgesic point in the body and is intensively stimulated in all painful conditions and is located on the highest point of the adductor pollicis muscle with the thumb and index finger adducted. “*Shousanli*” (LI 10) is located on the radial side of the dorsal surface of the forearm at about 3 cm below the lateral transverse elbow crease and between the extensor carpi radialis longus and brevis. “*Quchi*” (LI 11) is located on the end of the lateral transverse elbow crease at the middle of the connection between the biceps tendon and the lateral epicondylus of the humerus.

Subjects in the local acupuncture point (LP) group received acupressure at three tender points on the neck/shoulder muscles, which were consistent with local acupuncture points, “*Jianjing*” (GB 21), “*Jianwaishu*” (SI 14), and “Jianzhongshu” (SI 15) (Figure 1), subjects in the distal acupuncture point (DP) group received acupressure at three distal acupuncture points, “*Hegu*” (LI 4), “*Shousanli*” (LI 10), and “*Quchi*” (LI 11) (Figure 2), and subjects in the control group did not receive any stimuli.

All measurements were performed during the afternoon hours. Subjects were assessed regarding pain intensity using verbal rating scale (VRS), pain-related disability using Neck Disability Index (NDI), pain-related anxiety using State-Trait Anxiety Inventory-I (STAI-I), muscle hardness (MH) on bilateral trapezius muscles, pain-associated stress using salivary alpha-amylase (sAA) activity, heart rate variability (HRV), and satisfaction using VRS due to acupressure. For the VRS, the intensity of neck pain or stiffness was evaluated on a numerical scale from 0 to 3 (0: no pain, 1: mild pain, 2: moderate pain, and 3: severe pain). NDI, which was published by Vernon in 1991, is the most commonly used and validated scale designed to assess self-rated disability in patients with neck pain and disorder [13]. MH was evaluated using a tissue hardness meter (PEK-1, Imoto Machinery Co. Ltd., Kyoto, Japan) bilaterally on the midpoint between the spinous process of seventh cervical vertebra and the acromion. This point is located on the trapezius muscles, and the tender point of neck pain often lies on this point, which is just the acupuncture point, “*Jianjing*” (GB 21) [1]. sAA was evaluated using a hand-held sAA monitor (CM-2.1, Nipro, Osaka, Japan) [14]. Satisfaction due to acupressure was evaluated on a numerical scale from 0 to 3 (0: no satisfaction, 1: mild satisfaction, 2: moderate satisfaction, and 3: sufficient satisfaction). VRS and STAI-I before, immediately following, and 1 day after receiving the treatment, MH and sAA before and immediately after the treatment, NDI before and 1 day after the treatment, satisfaction immediately following and 1 day after the treatment were sampled.

After the initial assessment, the subjects were allowed to lie comfortably on the bed in a quiet environment for 5 min. Then, the record of the electrocardiogram (ECG) signals for HRV analysis started. 

Ten minutes later, three sets of acupressure by the pulp of the right thumb in a rotary fashion at 20–25 cycles per minute for 30 seconds on each point were administered at the right side of GB 21, SI 14, and SI 15 consecutively and afterwards at the left side of these three points in the LP group. On the other hand, three sets of procedures conducted in the same way as shown in the LP group were administered at the right side of LI 4, LI 10, and LI 11 consecutively and afterwards on the left side of these three points in the DP group. These procedures were applied by the same investigator. Following release of acupressure, the subjects were observed for another 10 minutes. The ECG signals were obtained from a portable ECG (AC301A, GMS, Tokyo, Japan) and transferred to a computer loaded with HRV analysis software (TARAWA/WIN; Suwa Trust, Tokyo, Japan). The R-R intervals (RRIs) were obtained every 10 seconds. The two components of power of the RRI (ms.ms), LF (0.04–0.15 Hz) and HF (0.15–0.5 Hz), were calculated. Heart rate (HR) and the LF and the HF values and the LF/HF ratio of HRV were analyzed. The data of HR and HRV values for 30 seconds at 5 minutes before the beginning of the pressure (pre-treatment) and for 30 seconds at 5 minutes after pressure release (post-treatment) were sampled for subsequent analysis.

### 2.3. Data Analysis

Data was presented as mean (SD). VRS, STAI-I, MH, NDI, HR, and HRV values were analyzed with Kruskal-Wallis test for intergroup comparison followed by Dunn's Multiple Comparison Test. Satisfaction due to acupressure was analyzed with Mann-Whitney's U test for intergroup comparison on the LP and the DP groups. VRS and STAI-I were analyzed using Friedman test for intragroup comparison followed by Dunn's Multiple Comparison Test. Wilcoxon signed-rank test was used to analyze MH, NDI, HR, and HRV values for intragroup comparison. *P* < .05 was considered as statistically significant.

## 3. Results

Table 1 shows the demographic data of the three groups. There were no significant differences in age, weight, and pre-treatment values regarding pain conditions among the three groups (Table 1).

**Figure 3:**
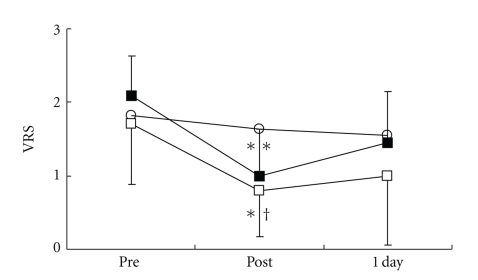
Changes in pain intensity (VRS: verbal rating scale). ○: control group. ■: local acupuncture point (LP) group. □: distal acupuncture point (DP) group. Values are presented as mean. SD represented with error bars in the LP and the DP groups. * significantly different from pre-treatment in the DP group (*P* < .05). ** significantly different from pretreatment in the LP group (*P* < .01). † significantly different from control group in the DP group (*P* < .05).

**Figure 4:**
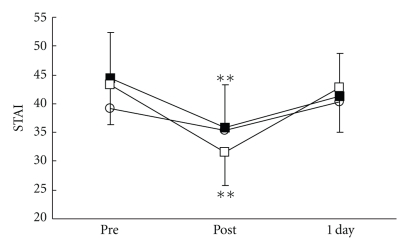
Changes in pain-associated anxiety (STAI-I: State-Trait Anxiety Inventory-I). ○: control group. ■: local acupuncture point (LP) group. □: distal acupuncture point (DP) group. Values are presented as mean. SD represented with error bars in the LP and the DP groups. ** significantly different from pre-treatment in the LP and the DP groups (*P* < .01).

**Figure 5:**
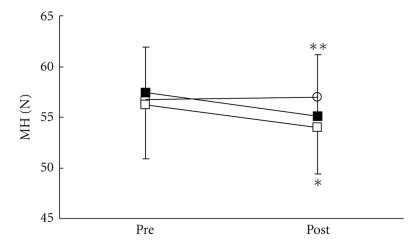
Changes in muscle hardness (MH). ○: control group. ■: local acupuncture point (LP) group. □: distal acupuncture point (DP) group. Values are presented as mean. SD represented with error bars in the LP and the DP groups. * significantly different from pre-treatment in the DP group (*P* < .05). ** significantly different from pre-treatment in the LP group (*P* < .01).

**Figure 6:**
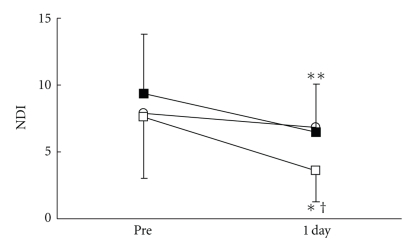
Changes in pain-associated disability (NDI:Neck Disability Index). ○: control group. ■: local acupuncture point (LP) group. □: distal acupuncture point (DP) group. Values are presented as mean. SD represented with error bars in the LP and the DP groups. * significantly different from pre-treatment in the DP group (*P* < .05). ** significantly different from pre-treatment in the LP group (*p* < .01). † significantly different from control group in the DP group (*P* < .05).

**Figure 7:**
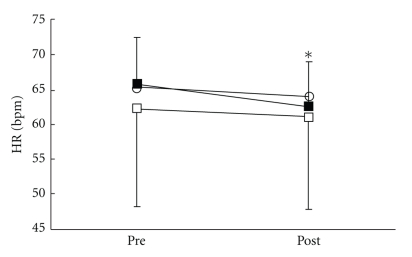
Changes in heart rate (HR). ○: control group. ■: local acupuncture point (LP) group. □: distal acupuncture point (DP) group. Values are presented as mean. SD represented with error bars in the LP and the DP groups. * significantly different from pre-treatment in the LP group (*P* < .05).

**Figure 8:**
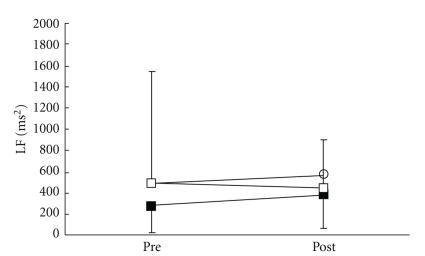
Changes in the low-frequency (LF) component of heart rate variability. ○: control group. ■: local acupuncture point (LP) group. □: distal acupuncture point (DP) group. Values are presented as mean. SD represented with error bars in the LP and the DP groups.

**Figure 9:**
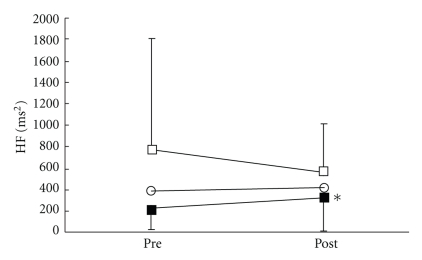
Changes in the high-frequency (HF) component of heart rate variability. ○: control group. ■: local acupuncture point (LP) group. □: distal acupuncture point (DP) group. Values are presented as mean. SD represented with error bars in the LP and the DP groups. * significantly different from pre-treatment in the LP group (*P* < .05).

**Figure 10:**
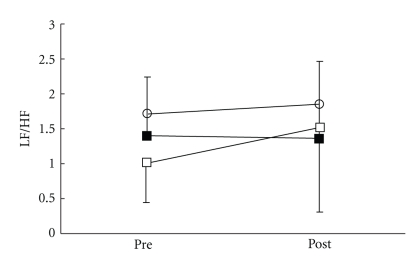
Changes in the LF/HF ratio (LF/HF) of heart rate variability. ○: control group. ■: local acupuncture point (LP) group. □: distal acupuncture point (DP) group. Values are presented as mean. SD represented with error bars in the LP and the DP groups.

**Figure 11:**
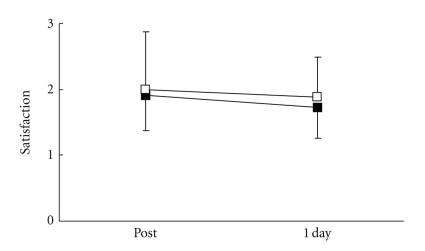
Changes in satisfaction due to treatment (VRS: verbal rating scale). ■: local acupuncture point (LP) group. □: distal acupuncture point (DP) group. Values are presented as mean. SD represented with error bars in the LP and the DP groups.

There were no significant differences in all parameters in the control group. VRS (Figure 3), STAI-I (Figure 4), and MH (Figure 5) values significantly decreased immediately after treatment, and NDI (Figure 6) was lower at 1 day following treatment compared with pre-treatment in the LP and the DP groups. HR (Figure 7) significantly decreased and the HF component of HRV (Figure 9) significantly increased after treatment in the LP group only. There were no differences on the sAA and the LF components (Figure 8) and the LF/HF ratio (Figure 10) of HRV among the three groups. Satisfaction due to acupressure continued to 1 day after the treatment in the LP and the DP groups (Figure 11).

## 4. Discussion

Our results demonstrated that acupressure on the local and the distal acupuncture points significantly reduced various parameters of the pain-associated conditions, that is, VRS, STAI-I, MH, and NDI whereas there were no significant differences in all parameters in the control group. Although acupressure did not change the LF and the LF/HF ratio of HRV, acupressure on the local acupuncture points significantly reduced HR and increased the HF of HRV. Satisfaction due to acupressure continued until 1 day after treatment on the distal points as well as the local points. These results show that acupressure on not only the local points but also the distal acupuncture points improved pain-related condition, and furthermore acupressure could influence the autonomic nervous system.

Mechanical pressure such as massage and acupressure has been known to decrease tissue adhesion, promote relaxation, increase regional blood circulation, increase parasympathetic nervous activity, increase intramuscular temperature, and decrease neuromuscular excitability [15]. Also, many researchers have demonstrated the effect of acupressure and acupuncture for sedation [4, 5, 16, 17]. 

Acupuncture on the tender points has been commonly used as a treatment for chronic neck pain and appears to alleviate pain and stiffness [1, 18]. The tender points are known to be located at traditional acupuncture points, “*ah si*” point, and also to conform with trigger points and criterion sites for fibromyalgia [1, 18, 19]. Tender points are supposed to be the site where there are nociceptors and polymodal receptors, which have been sensitized by various factors. Thus, stimulation such as acupuncture and acupressure on the tender points may activate sensitized polymodal receptors more powerfully, resulting in stronger effects on pain relief [1]. In traditional acupuncture medicine, tender points eliciting tenderness or pain could be selected when treating chronic neck pain [1]. 

Acupuncture treatment typically applies to not only the tender points but also the distal acupuncture points for the treatment of chronic pain. Acupuncture at the distal acupuncture points could improve pain conditions in chronic neck pain patients, indicating that nonsegmental antinociceptive systems may play a major role in acupuncture analgesia [2]. Also, electroacupuncture at the acupuncture point *“Hegu” *(LI 4) decreases the activity on anterior cingulated cortex (ACC) and cingulum, thereby inhibiting nociceptive processing in the brain. Acupuncture point stimulation at a rich nerve junction such as *“Hegu”* may reduce pain-induced cingulation processing, thereby resulting in pain relief/analgesia [20]. A study showed that acupuncture improved pain-related disability assessed by NDI [21], as observed in the present study. Furthermore, acupuncture may improve activities at work, the quality of sleep and consequently tiredness, pain-related quality of life, and psychological variables for women with chronic neck pain [22].

Acupuncture has been reported to affect the autonomic nervous system [11, 23]. However, acupuncture/acupressure might have different physiological effects between local and distal acupuncture points, since we showed that acupressure at LI-4, LI-10, and LI-11 did not, but at GB-21, SI-14, and SI-15 significantly influenced autonomic nervous activity.

There are several limitations to the present study. One of them is that we did not perform longer term followup after acupressure. We need further evaluation of the longer effects of acupressure on chronic neck pain and autonomic nervous system. Another limitation is that we showed only the effect of acupressure on either local or distal points. Most acupuncturists and acupressurists use both local and distal points together in clinical practice. Therefore, further study is required in order to assess combinational effects.

In conclusion, acupressure significantly improved pain conditions on not only the local points but also the distal acupuncture points in females with chronic neck pain but affected the autonomic nervous system on only local acupuncture points, as acupuncture points *per se* have different physical effects depending on location.

## Figures and Tables

**Table 1:** Age, weight, VRS, NDI, STAI, MH, sAA, HR, and HRV values at pretreatment for each group.

	C group (*n* = 11)	LP group (*n* = 11)	DP group (*n* = 11)	*P* value
Age (yr)	34.8 (4.0)	35.5 (6.4)	37.2 (7.0)	.637
Weight (kg)	50.4 (6.8)	52.3 (10.1)	52.2 (4.8)	.643
VRS	1.8 (0.6)	2.1 (0.5)	1.7 (0.8)	.413
NDI	7.9 (3.8)	9.4 (4.4)	7.6 (4.6)	.430
STAI	39.2 (9.5)	44.5 (8.0)	43.2 (6.8)	.772
MH (N)	56.9 (5.0)	57.4 (4.5)	56.2 (5.3)	.507
sAA (kU/l)	38.2 (20.1)	20.0 (9.0)	36.8 (27.9)	.079
HR (bpm)	65.4 (8.7)	65.8 (6.7)	62.3 (14.1)	.941
LF (ms^2^)	490.7(409.2)	274.2 (253.3)	494.1 (1050.7)	.084
HF (ms^2^)	381.8(338.3)	212.8 (186.7)	764.8 (1045.2)	.587
LF/HF	1.7(1.4)	1.4 (0.8)	1.0 (0.6)	.399

Values expressed as mean (SD).VRS: verbal rating scale. NDI: Neck Disability Index. STAI: State-Trait Anxiety Inventory-I. MH: muscle hardness. sAA: salivary alpha-amylase. HR: heart rate. LF: the power of low-frequency (0.04–0.15 Hz, LF) component of heart rate variability (HRV). HF: the power of high-frequency (0.15–0.4 Hz, HF) component of HRV. LF/HF: LF/HF ratio of HRV.
